# Seroprevalence of SARS-CoV-2 antibodies in Bali Province: Indonesia shows underdetection of COVID-19 cases by routine surveillance

**DOI:** 10.1371/journal.pgph.0000727

**Published:** 2022-08-31

**Authors:** Anak A. S. Sawitri, Putu C. D. Yuliyatni, Putu A. S. Astuti, Emita Ajis, Endang B. Prasetyowati, Husni, Juliette Morgan, Jennifer Mika, Catharina Y. Praptiningsih, Amalya Mangiri, Ester Mulyadi, Rintis Noviyanti, Leily Trianty, William A. Hawley

**Affiliations:** 1 Department of Public Health and Preventive Medicine, Faculty of Medicine, Universitas Udayana, Denpasar, Bali, Indonesia; 2 Directorate of Health Survaillance and Quarantine, Ministry of Health Republic Indonesia, Jakarta Indonesia; 3 Gedung Adhyatma Kementerian Kesehatan Republik Indonesia, Jakarta, Indonesia; 4 Indonesia Field Epidemiology Secretariate, Jakarta Pusat, Indonesia; 5 US Centers for Disease Control and Prevention, Jakarta, Indonesia; 6 Eijkman Institute for Moleculer Biology, Kota Jakarta Pusat, Daerah Khusus Ibukota Jakarta, Indonesia; University of Sri Jayewardenepura, SRI LANKA

## Abstract

The international tourist destination of Bali reported its first case of Coronavirus Disease 2019 or COVID-19 in March 2020. To better understand the extent of exposure of Bali’s 4.3 million inhabitants to the COVID-19 virus, we performed two repeated cross-sectional serosurveys stratified by urban and rural areas. We used a highly specific multiplex assay that detects antibodies to three different viral antigens. We also assessed demographic and social risk factors and history of symptoms. Our results show that the virus was widespread in Bali by late 2020, with 16.73% (95% CI 12.22–21.12) of the population having been infected by that time. We saw no differences in seroprevalence between urban and rural areas, possibly due to extensive population mixing, and similar levels of seroprevalence by gender and among age groups, except for lower seroprevalence in the very young. We observed no difference in seroprevalence between our two closely spaced surveys. Individuals reporting symptoms in the past six months were about twice as likely to be seropositive as those not reporting symptoms. Based upon official statistics for laboratory diagnosed cases for the six months prior to the survey, we estimate that for every reported case an additional 52 cases, at least, were undetected. Our results support the hypothesis that by late 2020 the virus was widespread in Bali, but largely undetected by surveillance.

## Introduction

Coronavirus Disease 2019 or COVID-19 is an infectious disease caused by severe acute respiratory syndrome coronavirus 2 (SARS-CoV-2) [1]. Indonesia reported its first COVID-19 case on 2 March 2020 in Jakarta [2, 3] with the international tourist destination of Bali reporting its first case shortly thereafter [4]. Reported COVID-19 cases in Indonesia generally increased throughout 2020 with some fluctuations [5]; similar trends were observed in Bali Province [6].

Published models from February 2020 suggested that COVID-19 reported cases in Indonesia were artifically low due to insufficient surveillance [7, 8] and imperfect reporting associated with a surveillance information system [9] that sometimes failed to report detected COVID-19 cases to provincial authorities. Early in the pandemic, Indonesian epidemiologists noted the need to increase testing for better tracking and control of the virus [10]. In May 2020, testing in Indonesia was limited, with only 225 tests administered per 1 million people, or 5.6% of the testing needed based upon projections at the time [10]. The problem of low testing was further compounded by inadequate tracking of confirmed cases, whereby data from the Bali Province Health Office showed that the average number of contacts traced per case was only nine persons in 2020, declining to less that 5 persons per case in October 2021 [11]. Thus, the true extent of virus spread in Bali was poorly understood.

Underreporting is exacerbated by the fact that most of Bali’s reported cases were from hospitals treating symptomatic patients, which represent only a fraction of the clinical spectrum. The importance of pre-symptomatic COVID-19 transmission is well-documented whereby 44% of secondary cases were contracted before onset of COVID-19 symptoms [12]. The distinction between asymptomatic and pre-symptomatic transmission is problematic, whereby ‘true’ asymptomatic patients may be early in the process of developing symptoms [13]. A systematic review and meta-analysis revealed that asymptomatic persons have 50% lower probability to transmit SARS-CoV-2 compared to those with symptoms [14].

In addition to asssessing the extent of exposure, seroprevalance surveys are useful for monitoring trends, geographic distribution, and targeting interventions [15]. At the time of this survey, similar seroprevalence studies had been reported elsewhere [16, 17] but none had been conducted in Bali. Further, our repeated cross-section study design has the potential to assess the rapidity of spread of the virus. Our expectation is that our results will inform public health measures for better surveillance and control [15].

## Material and methods

### Ethics and permission

Ethical approval was received from the Ethics Committee of Faculty of Medicine Udayana University on 20 July 2020 prior to the first round of the survey, and an amendment was proposed for measurement of blood pressure and blood sugar for adults and determining blood type for children as incentives for round 2; approval for the amendment was granted on 7 November 2020. Written permission from the Governor of Bali Province had been granted before the data collection. All survey procedures and related information were explained to eligible subject or their legal representative, by enumerators on behalf of principal investigator prior the data collection. We interviewed and took blood samples only from parents and eligible respondents who provide their written consent.

### Setting and study design

Bali Province has a population of 4.3 million with 785,000 people in its capital, Denpasar [18]. Population mobility is high in and out of Bali Province, between districts, and between urban and rural areas [19]. For this survey, Denpasar, with a population density of 6,360 people/km2 was considered an urban area, while the remainder of the island with a population density around or below of 7.50 people/km2 was considered rural [20].

Cross-sectional sero-surveys were conducted in two rounds between 14 October– 24 December 2020. Specimen collection for round 1 occurred from 14 October– 14 November and for round 2 from 15 November– 24 December. Urban (Denpasar) compared to rural (outside Denpasar) stratification was done. Multi-stage cluster random sampling of census enumeration areas was performed.

### Population and sample

#### Sample size and power

The survey was designed to provide an estimate of seroprevalence of SARS-CoV-2 in Bali Province, in urban Denpasar, and in rural areas outside of the capital. The minimum required sample size was calculated using this formula.


$$Minimum\;required\;sample\;size=DE\times \frac{Z_{1-\alpha /2}^{2}\times P\times (1-P)}{d^{2}}$$

We assumed that the expected prevalence of antibodies to SARS-CoV-2 was higher in Denpasar (7%) than in the rest of the island (5%). We also assumed a conservative design effect (DE) of 2 to account for an expected increase of variance due to clustering effect, a response rate of 75%, and a desired confidence level of estimate _1-α/2_ = 95% (Z_1-α/2_ = 1.96) and a 2% margin of error (d), resulting in a sample size of 1,555 in urban Denpasar and 1,138 in rural areas (total of 2,693 individuals) per survey. With an average expected number of persons per household of 3.46 in Denpasar and of 3.89 in the non-Denpasar areas [21], the number of households expected to be visited was 450 in urban and 293 in rural areas (total of 743 households) per survey.

#### Sample, household (HH) and participant selection

A household was defined as a group of persons who reside in the same place and prepare meals together. All members of selected households who were ≥1 year of age were eligible if informed consent was obtained by either the participant of the parent or guardian.

The National Statistics Office provided enumeration areas (census blocks based upon a March 2020 national social and economic survey) for Bali as the sampling frame. Twenty HHs were selected per census block, with 23 from Denpasar and 15 from rural districts, for a total of 38 blocks.

A two-stage systematic random sampling strategy was applied. In stage 1, census blocks were selected in urban and rural areas, while in stage two, 20 HHs in each census block were selected after performing stratification by educational level of the head of HH. The samples were mutually independent between surveys, whereby the census blocks with odd serial numbers were used as the sampling frame for the round 1 survey, while census blocks with even serial numbers were used for round 2. A sample reserve of 20% or 4 HHs per census block was prepared. Selection of backup samples was carried out by systematic sampling from the list of HHs not selected in the original procedure.

### Data collection

#### Training

A technical guide for fieldwork including all survey procedures was prepared and reviewed during two days of training for the eight survey teams. The survey teams were monitored by project staff throughout the survey.

#### Questionnaire

A structured questionnaire in the Indonesian language was developed based on previous behavioral and risk factor assessments for SAR-CoV2 exposure [22–24]. Basic demographic and economic information (number of HH members, HH income, age, gender, education, and marital status) was obtained along with a history of symptoms during the last 6 months. We also developed a non-response form for refusers. The interview questions were pilot tested and evaluated by local experts to ensure that they were culturally appropriate and could be understood by a layperson with a primary education. The questionnaire was revised after piloting in enumeration areas not selected for the survey.

#### Interview

Survey teams visited HHs in coordination with local leaders and guides from the district statistical office. Interview responses were recorded on a password protected hand-held device that uploaded to a secure database upon completion of the questionnaire. Paper format was used in areas where internet access was limited.

#### Blood sampling and processing

Dried blood spot (DBS) samples were collected by finger prick and spotted onto Whatman 903 filter paper. An identification number was placed on each filter paper. Each participant’s data included a unique identifier (barcoded label). Data collection in each census block was on average completed in four days.

DBS specimens were registered in an electonic log book for tracking. Blood spots were dried overnight at room temperature until uniformly dark brown with no red color visible. The DBS were then stored and shipped at 4–8°C via air to the Eijkman Institute for Molecular Biology in Jakarta, where they were stored at 20°C.

SARS-CoV-2 antibody testing was performed on a Luminex MAGPIX instrument using the Tetracore FlexImmArray SARS-CoV-2 Human IgG Antibody assay [25]. This test detects antibodies to three different antigenic sites on the virus: spike (S), nucleoprotein (NP) and hybrid using a Multiplex Bead technology. The test uses 7 microspheres, with three detecting antibodies to different SARS-CoV-2 antigens, while 4 are internal controls. Every 96-well test plate was used for testing up to 90 samples in one test run. Each test run included SARS-CoV-2 negative control serum, positive control serum, and calibrator in duplicates. Antibody response was determined qualitatively using the ratio of mean fluorescence intensity (MFI) target antigen/MFI calibrator. Results were considered SARS-CoV2 human IgG positive if all three target antigens had a ratio of ≥1.2, negative if the ratio was ≤0.9, and indeterminate if between this range. Specimens with indeterminate results were re-tested; if the second test was indeterminate the specimen was classified as negative. A positive result implied past exposure to the virus.

### Data analysis

Weighting was performed by the National Statistical Office to account for sampling design, response rate, and stratification. Descriptive analysis was conducted for characteristics of the HHs and respondents for round 1, round 2 and combined rounds. The overall prevalence of SARS-CoV-2 antibodies was calculated using weighted and unweighted data, per round and for both rounds combined. To estimate a population value, we calculated a range of estimates as a percentage, whereby a population mean lies between an upper and lower interval. Prevalence was calculated for rural and urban areas, by socio-demographic parameters, and by presence or absence of reported symptoms. Sensitivity analysis was not carried out, as an unpublished report showed that the Tetrocore test kit had a sensitivity of 89% and specificity of 100% [25]. Raw data used for analysis is provided in (S1 Data).

In Bali province, monitoring of COVID-19 cases is performed through active and passive surveillance systems. The passive system records the number of confirmed infections, deaths, and recovered hospitalized COVID-19 people from public health centers, local and central public hospitals, and some private hospitals in Bali Province. Meanwhile, active surveillance records cases found from contact tracing and screening at the entry points such as the airport and harbour. Information from active and passive surveillance is entered to the single sign on (SSO) system. The system was developed in mid June 2020 and was used until September 2021. Since then, COVID-19 data was transfered to a central information system called ‘New All Record’ (NAR). The New All Record system was established in April 2021 [26]. The possibility of a gap between detected and reported COVID-19 persons in this surveillance system is beyond the scope of our study.

Using the SSO data from 1 June—30 November 2020 (https://infocorona.baliprov.go.id/) for which period we assumed that infections would produce detectable antibodies during the time of our survey, we compared the estimated seroprevalence of SARS-CoV-2 with reported cumulative COVID-19 cases in Bali Province and calculated number of missed infections during this period.

### Results

Table 1 presents the characteristics of census blocks being surveyed. Among 38 targeted census blocks, one census block was not surveyed during round 1 due to refusal by local authorities; in round 2, all 38 census blocks were successfully surveyed. The acceptance rate of HHs and respondents was slightly higher in rural than in urban areas, and was higher in round 2 than in round 1. The acceptance rate for DBS collection was low in both rounds, but was higher in rural areas and in round 2. Very few samples failed quality control for the lab assays, with only 1.8% failing in round 1 and 1.1% failing in round 2. Samples that did not pass QC were excluded from the analysis.

**Table 1:** Characteristics of survey census blocks.

Districts	Census Block type			Description of Household			Respondent characteristics					Average number of respondents per HH
	Planned	Surveyed		Approached	Surveyed		Eligible HH members	Number interviewed		Blood Samples obtained		
	N	N	%	N	N	%	N	N	%	N	%	
**Round 1**												
**Bali**	38	37	97.4	793	742	93.6	2,392	2,353	98.37	1,175	49.94	3.22
**Urban**	23	23	100	504	463	91.8	1,396	1,362	97.56	591	43.39	3.01
**Rural**	15	14*	93.3	289	279	96.5	996	991	99.50	584	58.93	3.57
**Round 2**												
**Bali**	38	38	100	785	760	96.8	2,089	2,076	99.38	1,407	67.77	2.75
**Urban**	23	23	100	484	460	95.0	1,222	1,211	99.00	809	66.80	2.66
**Rural**	15	15	100	301	300	99.7	867	865	99.77	645	74.57	2.89

Note: Urban = Denpasar, Rural = 8 districts non-Denpasar; eligible member of HH: number of family member > = 1 years old plus non family member who stay in the same house, average respondents per HH: eligible member of HH/ selected HH Surveyed.

Table 2 presents the sociodemographic and household characteristics of respondents consenting to interview and blood sampling. Repondents in the 15–54 year old age group were more likely to consent to interview than those younger or older than this group. Gender and marital status were not associated with consent to interview. The median level of education was through high school, with mean household monthly income in both surveys about 3 million IDR (215 USD). While most households have ventilation, only 20% were airconditioned. Blood samples were obtained from a low proportion of children (1–4 y.o., and 5–14 y.o.) in both rounds, with an increase in round 2.

**Table 2:** Characteristics of survey participants.

Socio-demographic characteristics	Interviewed		Blood Sample obtained		Proportion of those interviewed with blood sample obtained	
	Round 1 (N = 2,351)	Round 2 (N = 2076)	Round 1 (N = 1,175)	Round 2 (N = 1,407)	Round 1	Round 2
	Freq (%)	Freq (%)	Freq (%)	Freq (%)	%	%
**Age Group**						
**1–4**	126 (5.36)	86 (4.14)	6 (0.51)	18 (1.28)	4.76	20.93
**5–14**	335 (14.25)	275 (13.25)	33 (2.81)	45 (3.20)	9.85	16.36
**15–24**	458 (19.48)	326(15.70)	63 (5.36)	87(6.18)	13.76	26.69
**25–34**	332 (14.12)	290(13.97)	98 (8.34)	108 (7.68)	29.52	37.24
**35–44**	366(15.44)	343 (16.52)	149 (12.68)	112 (7.96)	40.82	32.65
**45–54**	363 (15.44)	390(18.79)	85 (7.23)	96 (6.82)	23.42	24.62
**55–64**	223 (9.49)	207(9.97)	88 (7.49)	108 (7.68)	39.46	52.17
**65+**	148 (6.30)	159 (7.66)	97 (8.26)	114 (8.10)	65.54	71.70
**Sex**						
**Male**	1.171 (49.77)	1.033 (49.76)	572 (48.68)	651 (46.27)	48.85	63.02
**Female**	1.182 (50.23)	1.043 (50.24)	603 (51.32)	756 (53.73)	51.02	72.48
**Marital status**						
**Not married**	1,000 (42.50)	756 (36.42)	380 (32.34)	426 (30.28)	38.00	56.35
**Married**	1,310 (55.67)	1,262 (60.79)	772 (65.70)	929 (66.03)	58.93	73.61
**Divorce**	43 (1.83)	58 (2.79)	23 (1,96)	53 (3.70)	53.49	91.38
**Education**						
**Yet/never attended school**	265 (11.26)	181 (8.72)	73 (6.21)	91 (6.47)	27.55	50.28
**Not completed ES**	153 (6.50)	156 (7.51)	71 (6.04)	82 (5.83)	46.41	52.56
**Elementary school (ES)**	453 (19.25)	519 (25.00)	230 (19.57)	364(25.87)	50.77	70.13
**Junior high school**	345 (14.66)	298 (14.35)	202 (17.19)	226 (16.06)	58.55	75.84
**High school**	783 (33.28)	656 (31.60)	439 (37.36)	461(32.76)	59.90	70.27
**Diploma**	94 (3.99)	81 (3.90)	43 (3.66)	55 (3.91)	45.74	67.90
**Bachelor (D4/S1)**	240 (10.20)	174 (8.38)	106 (9.02)	123 (8.74)	44.17	70.69
**Master/Doctor**	20 (0.85)	11 (0.53)	11 (0.94)	5 (0.35)	55.00	45.45
HOUSEHOLD*	N = 740	N = 760				
**Household income (million IDR)**						
**Mean (SD)**	3,061 (4.18)	3.135 (6.28)				
**Range (million/month)**	0–54	0–88				
**Ventilation in house**	N = 740	N = 760				
**Available**	678 (91.62)	678 (89.21)				
**Not available**	62 (8.32)	82 (10.79)				
**Air conditioning in house**	N = 742	N = 760				
**Available**	166 (22.37)	147 (19.34)				
**Not available**	576 (77.63)	613 (80.66)				

*Characteristics of participating survey households

Table 3 shows details of seroprevalence for each survey round. The combined overall prevalence for both surveys (N = 2,545), without and with weighting were 17.5 (95% CI 16.01–18.96) and 16.73 (95% CI 12.22–21.12), respectively.

**Table 3:** Seroprevalence of SARS-CoV-2.

Variable	Round 1 (N = 1,154)				Round 2 (N = 1,391)			
	N total	%	95% CI		N total	%	95% CI	
			Lower	Upper			Lower	Upper
**Overall Prevalence**								
**without weighting**		16.72	14.57	18.88		18.12	16.09	20.14
**with weighting**		18.04	9.98	26.10		15.71	11.15	20.28
**Area**								
**Urban**	583	13.08	8.61	17.55	773	17.67	12.16	23.17
**Rural**	571	19.13	8.33	29.92	618	15.26	9.25	21.26
**Sex**								
**Male**	562	17.79	9.02	26.55	648	15.09	8.66	21.52
**Female**	592	18.29	9.94	26.63	743	16.28	11.50	21.06
**Age group (year)**								
**1–4**	7	0	-	-	18	9.4	0.00	28.29
**5–14**	92	25.89	12.15	39.63	132	14.99	5.84	24.13
**15–24**	246	15.69	6.16	25.22	220	16.95	9.24	24.67
**25–34**	167	17.98	12.06	23.89	202	16.68	8.19	25.18
**35–44**	201	16.67	7.34	26.02	249	14.11	7.68	20.55
**45–54**	213	18.76	7.56	29.96	290	18.10	8.61	27.60
**55–64**	137	18.62	5.39	31.85	152	16.74	7.57	5.91
**65+**	91	18.39	2.11	34.69	1128	11.81	4.47	19.15
**Education level**								
**Iliterate/not complete ES**	142	20.58	8.87	32.28	167	15.55	4.59	26.51
**ES–High School**	854	17.02	10.03	24.00	1,041	16.59	10.97	22.20
**Diploma/University**	158	20.88	9.84	40.85	183	9.66	2.52	16.81
**Marital satus**					N = 1,350*			
**Not married**	374	18.14	10.45	25.82	421	15.06	8.91	21.21
**Married**	758	18.40	9.9.16	27.65	918	16.03	10.13	21.95
**Divorced**	22	5.06	0.02	12.11	11	16.25	5.68	26.82
**Working status**	N = 1,153*							
**Unemployed**	522	18.02	8.89	27.23	625	13.39	8.64	18.15
**Employed**	631	18.06	10.00	26.03	766	17.84	11.43	24.24
**Symptom status**	N = 1,140*				N = 1,384*			
**No symptom**	919	16.20	9.86	22.53	1,091	13.72	8.99	18.45
**Common symptoms (fever, cough, rhinitis, dyspnea)**	100	23.23	6.54	39.91	117	20.65	7.72	33.57
**Less common symptoms (others)**	75	27.30	1.21	53.40	135	20.00	7.58	32.44
**With symptoms (less/common)**	46	34.76	7.00	62.50	41	26.45	8.35	44.55

Note: Seroprevalence of SARS-CoV-2 based on area, gender, sex, marital, education and symptoms are weighted; *contain missing data

Contrary to expectation, neither survey round showed a statistically significant difference in prevalence between urban versus rural areas. Prevalence based on socio-demographics also showed no significant difference between sex, age group, education level, marital and employment status in either survey round. Respondents with symptoms were more likely to be seropositive than asymptomatic individuals.

**Fig 1:**
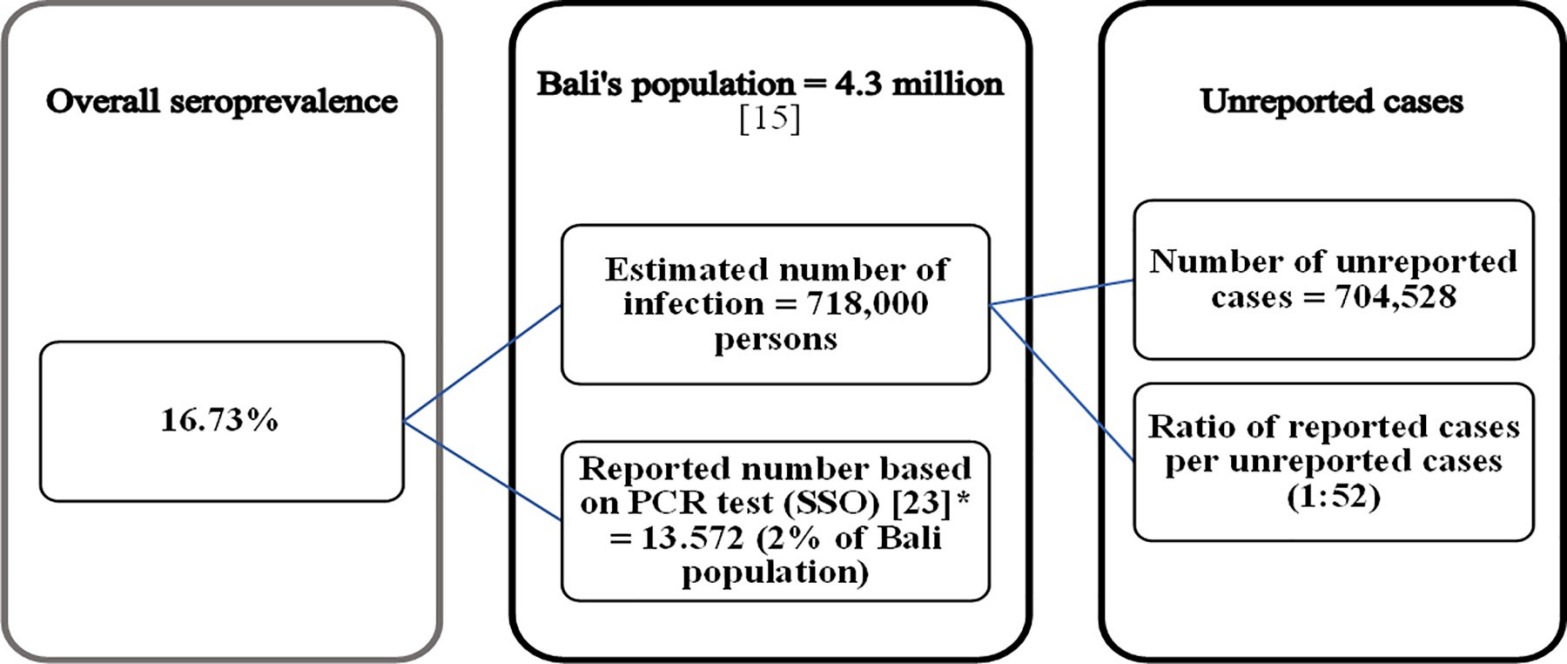
Comparison of reported case and estimated cases of SARS-CoV-2 infection based on serosurvey results.

As shown in “Fig 1”, seroprevalence results were extrapolated to estimate the total number of infections in Bali compared to to the actual number of infections reported by the surveillance system of the Government of Bali. The results show that only about 2% of infections were reported, with 52 cases likely occuring for each laboratory confirmed reported case.

Nearly three fourths of respondents reported no symptoms of SARS-CoV-2 infection in the last six months, as shown in Table 4. The most common symptoms reported were fever, headache, and rhinitis.

**Table 4:** Symptoms reported by indivuals positive for SARS-CoV-2 antibody within the last six months.

Symptoms	Round 1 (n = 192)		Round 2 (n = 250)		All Round (n = 442)	
	n	%	n	%	n	%
With symptoms						
No	140	72.9	180	72.0	320	72.4
Yes	52	27.1	70	28.0	122	27.6
Types of symptoms						
Fever	20	10.4	20	8.0	40	9.0
Rhinitis	10	5.2	13	5.2	23	5.2
Cough	10	5.2	11	4.4	21	4.8
Dyspnea	4	2.1	3	1.2	7	1.6
Sub-fever	3	1.6	13	5.2	16	3.6
Headache	13	6.8	21	8.4	34	7.7
Myalgia	7	3.6	4	1.6	11	2.5
Athralgia	11	5.7	4	1.6	15	3.4
Fatigue	11	5.7	2	0.8	13	2.9
Nausea	8	4.2	3	1.2	11	2.5
Vomiting	3	1.6	2	0.8	5	1.1
Diarrhea	3	1.6	5	2.0	8	1.8
Lose of appetite	4	2.1	1	1	5	1.1

## Discussion

This article reports results of a SARS-CoV-2 repeated cross-sectional serosurvey conducted in Bali Province in late 2020 [27]. The study was conducted relatively early in the pandemic, yet a high prevalence of SARS-CoV-2 antibodies was detected with a round 1 estimate of 18.04% (95% CI 9.98–26.10), a round 2 estimate of 15.71% (95% 11.25–20.28), and an overall combined prevalence of 16.73% (95% CI 12.22–21.12).

The results of this survey are similar to those obtained in July 2020 in India [16, 28], lower than results from a survey in Iran in April 2020 [29], but much higher than results from a systematic review for Asia and Southeast Asia for January–December 2020 showing seroprevalence of only 0.6% (0.3–1.4%) [27]. Within Indonesia, results from this survey were significantly lower than those obtained in Jakarta in March 2021, showing seroprevalence of 44.6% using the same laboratory methods [30]. However, the prevalence reported in the Bali survey was higher than that reported in during a comparable time in East Java (11%) [31].

The estimated level of SARS-CoV-2 prevalence from this survey implies that the surveillance system in Bali detected and reported an extremely low proportion of positive cases as shown in “Fig 1”. Meanwhile in Jakarta, a more massive testing effort was able to detect and report a higher proportion (8.1%) of infections [30]; however, this is still far below the required number by WHO [10, 11]. This condition occurred not only in Indonesia but elsewhere, as one systematic review has reported that numbers of infections estimated by serosurveys are always higher than actual number of cases detected [27]. The low proportion of positive cases detected by the surveillance system reflects the fact that neither Bali’s testing rate nor contact tracing efforts meet WHO standards [10, 11]. The weakness of surveillance systems to detect COVID-19 has also been reported in Africa [32]. Suboptimal reporting and monitoring of COVID-19 cases may create a false impression of decreasing of COVID-19 incidence [32]. Critical assessment of COVID-19 data in Indonesia [33] showed that the surveillance system only reported the confirmed, recovered and fatal cases and did not report suspected cases who died. It also missed the geographic and demographic details at the national, provincial and district level, as has been reported in Africa [32]. Suboptimal surveillance may mis-direct policy-making and control strategies [32] and may diminish the effectiveness of policy initiatives at the local level [33] and in the worst case adversely affect morbidity and mortality rates.

This survey was conducted using a standard methodology. Overestimation of prevalence due to clustering of cases within families was low, given that only 7% of families had more than one positive individual. As the laboratory test used requires positivity for antibodies to three different antigens, its specificity is high (100%) while sensitivity is 89% [15], leading to possible underestimation of seroprevalence.

Symptoms which were possibly related to COVID-19 were reported in 34.76% (95%CI 7.00–62.50) of respondents in round 1 and 26.45% (95% CI 8.35–44.45) of respondents in round 2. In contrast, seroprevalence in those not reporting symptoms was about half this level, though the differences were not statistically significant. In Iran, the proportion of seropositive asymptomatic individuals was much higher (57.2%) [17]. Even if the potential spread from asymptomatic cases is low [13], their relatively high numbers leads to significant transmission risk [34, 35], particularly in the context of weak active case detection and contract tracing.

Results from this study showed that 72% of seropositive respondents reported no symptoms whatsoever. Those numbers were probably too high [13] as a recent meta-analysis estimated the proportion of asymptomatic cases at 17% (95%CI 14–20%) [14], though several other studies showed wider variation [17, 36]. This study is subject to recall bias, as we asked about symptoms occurring in the past six months, whereby mild illness may have been forgotten or judged as not being ill at all. Further, at the time of the interview and DBS collection, some participants may have been subclinical or pre-symtomatic [14], which have shown high risk of transmission [13, 36]. Therefore, results from this survey support the conclusion that by focusing on symptomatic individuals, programs lose the opportunity to prevent transmission from asymptomatic individuals [13, 14].

No seroprevalence differences was found between the two survey rounds. During the first round of data collection, the response rate for blood draw was very low for various reasons (e.g. fear of diagnosis with COVID-19, fear of needles, too young for blood draw etc.). We attempted to increase the response rate by re-socialization to the mayor, head of districts and sub-districts, head of villages and sub-villages; and provided additional incentives, such as blood type test (for children), blood glucose test and blood pressure test (for adult respondents) for their participation. These measures helped to to increase the acceptance for blood draw in the second survey, resulting in a narrowing of the 95% confidence interval of SARS-CoV-2 seroprevalence in the second compare to first round survey. Nevertheless, analysis of sociodemographic characteristics of respondents for the two rounds showed that acceptance of blood draw for females was significantly higher than male (p 0.00), and for rural areas was significantly higher than for urban areas (p 0.00). Related to symptoms and acceptance for blood draw, the percentage of those reporting headache and dyspnea were higher in those providing blood samples compared those not reporting these symptoms. However, for other symptoms, we found no differences among those with and without symptoms, making us confident that differences in blood collection success between survey rounds did not result in significant bias.

We found no difference in prevalence between urban Denpasar and the rest of rural Bali. This may be because road transport and access throughout Bali is relatively good [13]. In addition, movement between rural and urban areas is common, as most respondents (round 1 = 71.9% and round 2 = 69.7%) reported participating in traditional ceremonies in their home villages while working or residing elsewhere.

This study has some weaknesses. First, response rate was low for DBS collection, with responses particularly low among very young participants. Second, as symptoms were reported by recall, the results are subject to bias. Nonetheless, results do show that the virus had spread substantially in Bali by late 2020, in contrast to findings from official statistics.

## Supporting information

S1 Data Data of seroprevalence of SARS-CoV-2 in Bali. (XLS)
